# Study of Cryogenic Unmasked Etching of “Black Silicon” with Ar Gas Additives

**DOI:** 10.1021/acsomega.1c06435

**Published:** 2022-02-08

**Authors:** Ekaterina A. Vyacheslavova, Ivan A. Morozov, Dmitri A. Kudryashov, Alexander V. Uvarov, Artem I. Baranov, Alina A. Maksimova, Sergey N. Abolmasov, Alexander S. Gudovskikh

**Affiliations:** † Alferov University (Saint Petersburg Academic University), 194021 Saint Petersburg, Russia; ‡ Saint Petersburg Electrotechnical University ≪LETI≫, 197376 Saint Petersburg, Russia; § R&D Center of Thin Film Technologies (Hevel LLC), 194021 Saint Petersburg, Russia

## Abstract

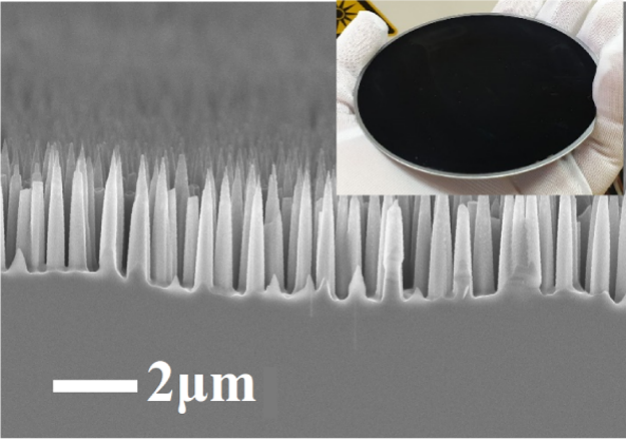

The influence of Ar gas additives on ≪black silicon≫ formation is shown in this work. The way to achieve the conical shape of Si texture using low Ar dilution is demonstrated. Also, a possibility of silicon nanowire width reduction keeping a high density of array is shown. No damage to the Si structure caused by Ar plasma was detected. The introduction of Ar into the plasma also does not affect electrical properties. The lifetime value after cryogenic etching with 5 sccm Ar flow remains at the same level of 0.7 ms. The resulting black silicon has a low total reflectance of 1 ± 0.5% in the range of 450–1000 nm in the overall 100 mm Si wafer surface.

## Introduction

Recently, there has been a transition from planar structures to three-dimensional ones. One of these types of structures is the structure of vertically aligned silicon (≪black silicon≫). Black silicon makes it possible to obtain solar cells with an efficiency close to 22%.^1^ Gas sensors based on black silicon have very high sensitivity and selectivity.^2^ Also, black silicon can be used for medical purposes.^3^ One of the main advantages of black silicon is its low reflection coefficient within a wide incident angle range. For the first time, black silicon was obtained as an etching artifact of the reactive ion etching (RIE) process.^4^ In 1997, the first solar cell based on black silicon with an efficiency of 17.1% was obtained using RIE.^5^ However, the RIE process leads to degradation of the charge carrier lifetime of the Si substrate.^6^ A modified RIE process, namely inductively coupled plasma (ICP) RIE, is believed to create lower defect density on the silicon surface.^7,8^ Further reduction of defect density could be achieved using the etching process at cryogenic temperatures.^9,10^ An additional advantage of cryogenic dry etching is that a black silicon surface could be produced without a mask.^11,12^ A black silicon surface formed by cryo-ICP etching with nanowires of 800 nm height and 200 nm diameter was used for IBC solar cells. A short-circuit current of 42.2 mA/cm^2^ was reached for such solar cells under AM1.5G with an EQE close to 100% in the wavelength range of 300–1000 nm being almost independent of the incidence angle. A total reflectance of 1% can be achieved using black silicon as the texture for the top contact of the solar cell. Microcrystalline n-type silicon solar cells obtained in this way demonstrated 21.9% efficiency.^13^ The rough surface of black silicon acts as recombination centers for minority charge carriers, which leads to a decrease in photovoltaic characteristics and, therefore, requires appropriate passivation. Excellent surface passivation can be achieved with amorphous hydrogenated silicon (a-Si:H), which provides a record low surface recombination rate of 0.7 cm/s.^14^ Also, well-known materials for surface passivation of silicon are silicon oxide (SiO_2_) and silicon nitride (SiN_*x*_).^15,16^ However, in the case of black silicon, the conformal growth of the passivation layers is an important issue. It is especially crucial for the solar cells based on the a-Si:H/c-Si heterojunction, where precise control of undoped a-Si:H layer thickness is required. Thickness uniformity could be much easily achieved for the conical shape of Si texture. Black silicon with such a cone-like shape has demonstrated an extremely high quantum efficiency in the short-wavelength region, showing evidence of effective passivation.^17^ Thus, finding ways to achieve Si wire shape control is quite important for further black silicon technology development.

To obtain vertically aligned structures on silicon, it is possible to use the processes of plasma etching in SF_6_/O_2_ gas at cryogenic temperatures, without applying a mask. In the process of plasma etching, the surface of the silicon substrate is bombarded with SF_*x*_ radicals (0 ≤ *x* ≤ 5), formed as a result of the dissociation of SF_6_ molecules, with the formation of volatile compounds SiF_*x*_ and SiF_*x*_O_*y*_. Under standard conditions, (at temperatures > −100 °C), the process exhibits isotropic etching behavior. With a decrease in temperature, condensation of SiF_*x*_O_*y*_ molecules occurs on the cooled surface of the silicon substrate, which creates a passivation layer on the silicon surface. Since in plasma etching, active SF_*x*_ radicals have a strict direction of motion under the action of the pulling plasma field, the vertical etching rate is much higher than the lateral one. At a sufficiently low temperature and a high oxygen content in the gas mixture, the mode of passivation of silicon and vertically aligned structures (≪black silicon≫) begins. The addition of inert gases makes it possible to increase the intensity of the ion bombardment of the etching bottom and therefore potentially change the shape of Si wires formed during the process. Argon (Ar) is widely used for the ion beam etching process.^18,19^ There are a few reports where argon was added for RIE of Si through a mask. Chen et al. reported about the effect of alternating Ar and SF_6_/C_4_F_8_ gas flows in Si plasma etching. The addition of the Ar step increased the etching rate and improved the selectivity of Si over a polymer mask.^20^ However, to the best of our knowledge, argon has not been used to form black silicon by cryogenic unmasked etching. The influence of Ar gas additives on black silicon formation is explored in this paper.

## Results and Discussion

**Figure 1:**
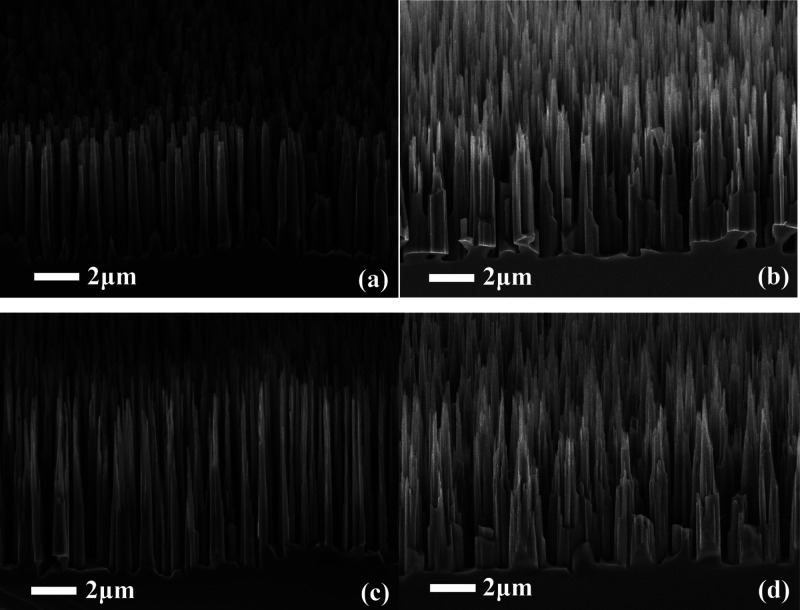
SEM image of etched black silicon during 440 s with an Ar flow of 0 (a), 2 sccm (b), 5 sccm (c), and 7 sccm (d).

The scanning electron microscopy (SEM) images of the Si surface obtained for the processes with a variation of Ar flow from 0 to 7 sccm are presented in Figure 1. For the process without Ar, a uniform array of Si nanowires (SiNWs) of about 5 μm height and 0.5–0.6 μm width is formed on the whole surface of Si wafers. The addition of Ar flow affects the shape of SiNWs. First, a rise of Ar flow up to 5 sccm leads to the decrease in the medium width of SiNWs down to 0.15–0.3 μm accompanied by a slight increase in the height (5.3 μm). A further increase in the Ar flow leads to the SiNW density decrease with the width rise. This phenomenon is probably caused by the fact that a high Ar content in the plasma leads to enhanced ion bombardment damage of the top silicon surface. The latter diminishes the over passivation effect of cryogenic etching. Ar is an inert gas and does not participate in the chemical etching reactions. Ar^+^ ions are extracted from the plasma by an electric field and bombard the substrate surface. When Ar^+^ ions collide with the substrate surface, energy is transmitted to the near-surface atoms of the material. Thereby, Ar^+^ ions affect the chemical reactions occurring at the Si surface.

**Figure 2:**
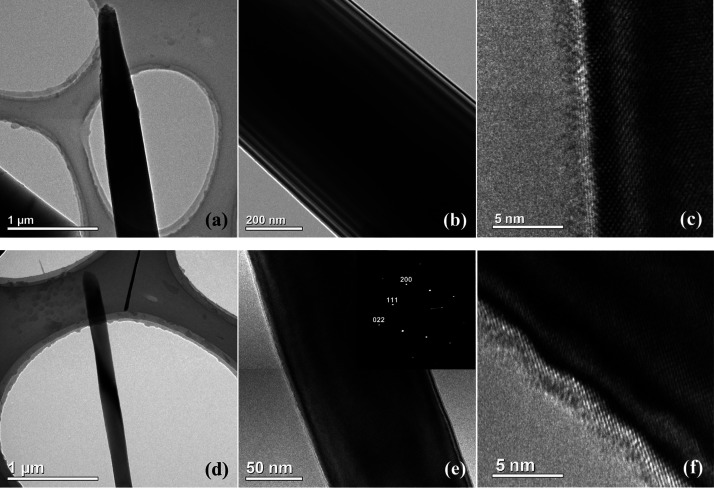
TEM image of SiNW obtained without (a–c) and with an Ar flow of 5 sccm (d–f).

However, weak dilution of the SF_6_/O_2_ mixture by Ar allows one to reduce the width of SiNWs while keeping a high density of array. On contrary, Ar plasma is known to have a destructive effect on Si. To study the effect of Ar dilution on the SiNW structural properties, they were explored by transmission electron microscopy (TEM) measurements. The TEM images of SiNWs obtained without and with a weak (5 sccm) Ar flow are presented in Figure 2. For both cases, a smooth wall side surface of SiNWs is observed (Figure 2a,b,d,e). The high-resolution TEM images (Figure 2c,f) demonstrate a perfect crystalline structure at the SiNW surface, which is covered by 3–5 nm of native oxide. No nanoporosity, amorphization, or any other structural defects were detected. Both SiNWs are of high crystalline quality and aligned along the [100] direction. The only difference between SiNWs obtained with and without an Ar flow is the width, which is significantly lower in the case of Ar dilution. Thus, no damage to the Si structure caused by Ar plasma was detected by TEM.

**Figure 3:**
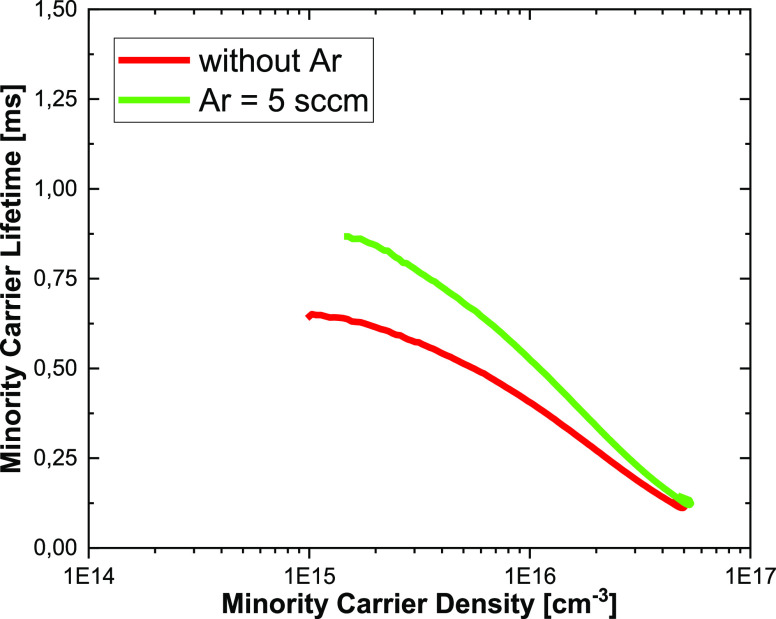
Minority carrier lifetime vs minority carrier density from QSSPC measurements.

The introduction of Ar into the plasma also does not affect electrical properties. This was confirmed by the minority charge carrier lifetime measurements performed for double-side polished Si wafers passivated by a-Si:H. The surface of polished wafers remains smooth after cryogenic etching with and without Ar. Thus, any influence of Si wire geometry is avoided. The lifetime value after cryogenic etching with a 5 sccm Ar flow determined by photoluminescence decay (PLD) remains at the same level of 0.7 ms as for the etching process without Ar plasma (0.5 ms). The PLD results correlate well with quasi-steady-state photoconductance (QSSPC) measurements (Figure 3), which confirm that the cryogenic process with Ar plasma could provide a minority charge carrier lifetime value slightly below 1 ms. This is a promising result for photovoltaic application of black silicon fabricated using such a process.

**Figure 4:**
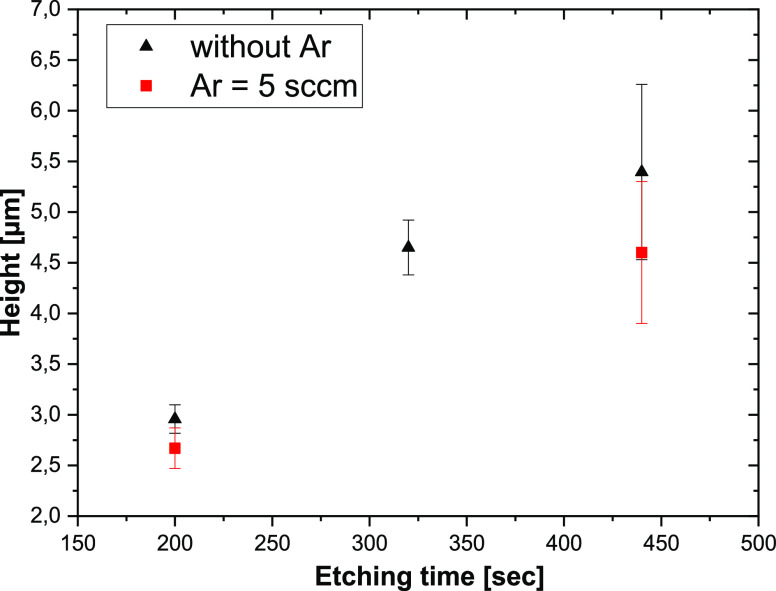
SiNW height as a function of etching time.

**Figure 5:**
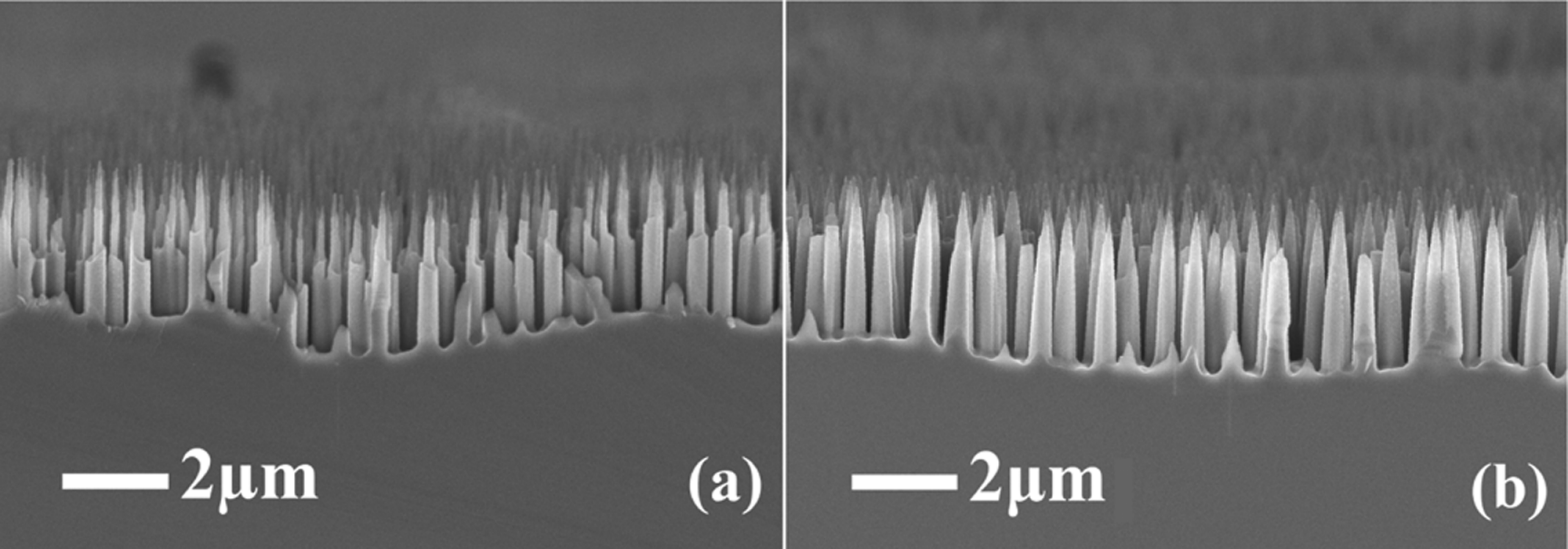
SEM image of etched black silicon during 200 s without Ar (a) and with 5 sccm Ar (b).

However, the size of SiNWs (Figure 1) with a high aspect ratio is not suitable for effective passivation being an important issue for solar cell performance. Lower height would be preferred to provide better passivation. The height of SiNWs could be reduced by lowering the etching time. The dependence of the SiNW height on the etching time is presented in Figure 4. The SiNW height increases with the etching time up to approximately 300 s, and then, the height is saturated at the value of about 5.5 ± 0.7 μm. The saturation occurs due to the etching of the pointed top of SiNWs during the process. Thus, the etching time could be significantly reduced to obtain the required height of SiNWs for photovoltaic application. The SEM images of the Si surface etched without Ar and with a 5 sccm Ar flow are shown in Figure 5. Despite the same height, SiNWs have different shapes. SiNWs etched without Ar have a pointed top shape, while in the case of Ar dilution, they are cone-shaped. The conic shape of SiNWs is much preferable in terms of passivation by further deposition of the passivation layer.

**Figure 6:**
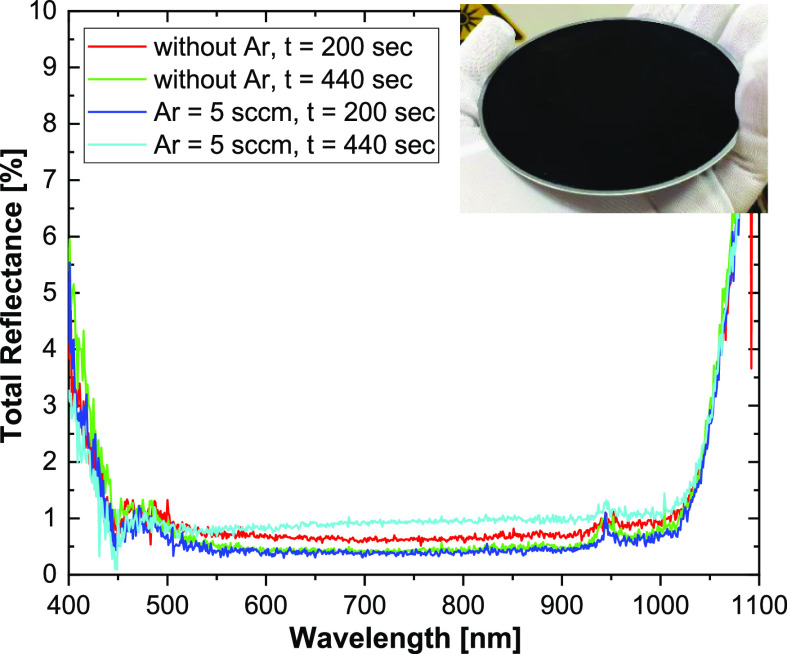
Total reflectance spectra for the Si wafers etched without and with an Ar flow (5 sccm) during 200 and 440 s. An array of SiNWs obtained on the entire 100 mm silicon substrate is presented in the inset.

However, the optical properties of the formed array of SiNWs are another important issue, which should be also verified. The total reflectance spectra of the SiNWs arrays with different heights and fabricated with and without Ar dilution are shown in Figure 6. All structures exhibit reflectance less than 1 ± 0.5% in the 450–1000 nm range. Thus, in terms of optical reflectance, the structures with a higher SiNW height (5–6 μm) have no practical advantage compared to those with a lower height of about 3 μm. Finally, a photo of a 100 mm silicon wafer etched with Ar dilution (inset of Figure 6) demonstrates the uniform distribution of the SiNW array on the entire substrate.

## Conclusions

The cryogenic unmasked etching of ≪black silicon≫ with Ar gas additives was studied in this work. Weak dilution of the SF_6_/O_2_ mixture by Ar allows one to reduce the width of SiNWs while keeping a high density of array. The cone-like shape of SiNWs could be achieved with Ar dilution being an important feature for precise control of passivation layer thickness. No structural defect caused by Ar plasma was detected by TEM. The lifetime value after cryogenic etching with a 5 sccm Ar flow remains at the same level of 0.7 ms as for the etching process without Ar plasma. In addition, the resulting black silicon has a low total reflectance of 1 ± 0.5% in the range of 450–1000 nm.

## Experimental Section

100 mm unpolished (100) n-type silicon substrates were used. The substrates were preliminarily cleaned in isopropanol and deionized (DI) water for 5 min to remove organic contaminants. The native oxide was not removed.

For the maximum effect of over passivation, cryogenic etching was performed at a temperature of −150 °C. The SF_6_ and oxygen content remained constant for each process with a flow of 15 and 45 sccm for O_2_ and SF_6_, respectively. The pressure of 5 mTorr and ICP power of 1000 W were fixed. The RF power of 40 W (88 mW/cm^2^) was used, which provides a −220 V DC bias. For the first series of processes, the argon flow was varied in the range from 0 to 7 sccm, while the etching time of 440 s was fixed. For the second series with 5 sccm Ar and without an Ar flow, the etching time was varied in the range from 200 to 440 s. We should stress that unpolished Si substrates should be used to achieve a uniform reproducible black silicon formation overall wafer surface.

The morphology of the resulting structures was studied using a SUPRA 25 Zeiss scanning electron microscope and TEM. TEM measurements were carried out using a Jeol JEM-2100 F microscope (accelerating voltage 200 kV, point resolution 0.19 nm). Specimens for TEM were prepared by the dry mechanical transfer of the wires onto a carbon lacey film supported by a copper TEM grid.

To estimate the influence of dry etching on photoelectrical properties, the charge carrier lifetime measurements were performed after passivation of polished Si wafers by a-Si:H. FZ double-side polished (100) n-Si wafers (5–10 Ohm cm) with a bulk charge carrier lifetime of about 10 ms were used. Dry etching of polished wafers does not lead to the formation of black Si. The surface remains smooth, allowing one to distinguish the influence of Ar plasma on the charge carrier lifetime independent of the morphology. After plasma etching, the wafers were cleaned by the Shiraki process.^21^ The native oxide was removed from the Si surface by HF dip prior to the a-Si:H deposition. Plasma-enhanced chemical vapor deposition (PECVD) of a-Si:H layers was performed on both sides of the Si wafer. PECVD was carried out at 250 °C from the SiH_4_ and H_2_ gas mixture at 350 mTorr and 11 mW/cm^2^ RF power density. This process was successfully used to achieve lifetime values of a few ms for polished Si wafers.

The charge carrier lifetime in the range of low minority carrier density (10^14^ to 10^15^ cm^–3^) was determined by PLD measurements described elsewhere.^22^ For the higher minority carrier density (10^15^ to 5 × 10^16^ cm^–3^), lifetime was detected by QSSPC measurements using a Sinton instruments WCT-120. The total reflectance spectra were also obtained using an integrating sphere and AvaSpec SensLine spectrometer.
